# Preparation of high permeable alumina ceramic membrane with good separation performance *via* UV curing technique

**DOI:** 10.1039/c7ra13195j

**Published:** 2018-04-11

**Authors:** Yang Liu, Weiya Zhu, Kang Guan, Cheng Peng, Jianqing Wu

**Affiliations:** School of Materials Science and Engineering, South China University of Technology Guangzhou 510640 People's Republic of China imjqwu@scut.edu.cn +86 20 87110273 +86 20 87111669

## Abstract

The traditional dip-coating method for preparation of ceramic membranes requires a long drying time and easily produces drying defects. In this work, an improved dip-coating process was proposed. The UV curing technique was utilized to avoid crack formation and agglomeration of ceramic particles, for drying to be completed in a few minutes. Photosensitive resin and a photoinitiator were added into the aqueous ceramic suspension. Under the action of free radicals excited by ultraviolet light, a giant network formed in the green membrane within a short time which limits the migration of membrane particles. Experiments were performed to explore the influence of UV curing process on membrane properties and the optimum preparation conditions were obtained. Following a rapid drying treatment and firing, crack-free membranes were prepared, which exhibited a narrow pore size distribution centered at approximately 65.2 nm and a water permeance of 887 ± 48 L m^−2^ h^−1^ bar^−1^. The largest pore size of the membrane was 85.7 nm while it could filter out 98.2% of the 100 nm monosize PS microsphere and the 60.1% of 60 nm, indicating its potential application in both membrane production efficiency and separation accuracy improvements.

## Abstract 2

The traditional dip-coating method for preparation of ceramic membranes requires a long drying time and easily produces drying defects. In this work, an improved dip-coating process was proposed.

## Graphical abstract

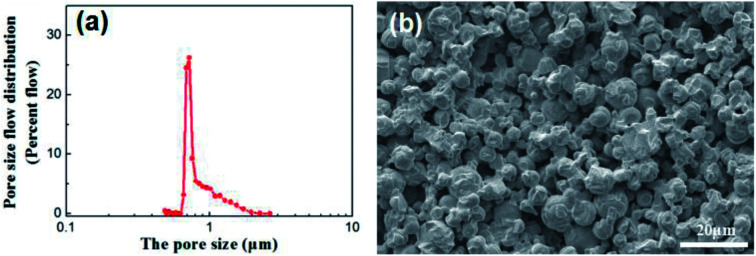

## 1. Introduction

In recent decades, porous ceramic membranes have been successfully used in many industry areas, such as waste solution treatment,^1^ oil concentration^2^ and gas separation process^3^*etc.* The formation of ceramic membranes through dip-coating on ceramic supports is a very common procedure to prepare micro- and ultra-filtration membranes.^4–7^ The crucial part of dip-coating process is the preparation of membrane-forming suspension which mainly consists of ceramic powders and other additives such as binders, dispersants and plasticizers. With higher environmental protection criteria implemented, aqueous membrane-forming suspension is becoming increasingly common for being eco-friendly and of low-cost compared to organic solvents. Adversely certain disadvantages are commonly confronted in a water based system, leading to the poor performances of the final products. These negative consequences include a long drying time and high crack sensitivity, due to the huge surface tension of water.^8^

Once a wet membrane containing suspended submicron-sized particles is coated on the porous support, the shrinkage occurs with the loss of water by evaporation. Further evaporation exerts compressive capillary force on the particle network under induction of surface tension.^9^ The support is free of contraction while the membrane generally binds to the support surface, which gives rise to the transverse tensile stresses. Cracks are formed spontaneously when the magnitude of the tensile stress exceeds a critical value.^10^

A commonly utilized method to reduce drying defects is to add membrane forming agents in the membrane-forming suspension. Researchers have studied the drying behavior of wet membranes with polymer binders coated on porous supports.^4,11,12^ The results demonstrated that a sufficient amount of reasonable polymer binder (such as PVA, PVP and MC) in the green membrane would contribute to avoid crack formation resulting from drying shrinkage.^4^ This was mainly due to the combination of particles with the polymer binder, induced by the hydrogen bonding of polymer binder molecules,^13^ which led to the membrane tensile strength improvement. Even though the addition of polymer binders may eventually form a crosslinking framework, the early strength of a wet membrane is not sufficient to resist the cracking under rapid dehydration. Therefore, the solvent removal rate is a fatal factor during drying, which is controlled through the ambient temperature and humidity adjustments. For this reason, a long drying time (almost beyond 12 h) and strict drying conditions are required at the initial stage of drying,^14–16^ which is a time-consuming and cumbersome process.

At present, the UV curing technology has been widely used in the coatings industry^17–19^ and in 3D printing^20^ due fact that it promotes fast polymerization and solidification of the prepolymer network. In addition to high production efficiency, the other advantages of light curing technology are high efficiency energy utilization as well as no solvent emission absence, signifying that it is safe, low-cost and pollution-free. Caroline Durif *et al.*^11^ demonstrated the possibility of solvent-free tape casting through UV curable binder. Unfortunately, few studies have been focused on UV curing process used in the preparation of ceramic membranes.

This paper reports a fast membrane process by adding appropriate amount of photosensitive resins and corresponding photoinitiators in the conventional membrane-forming suspension. The selected resins are water dispersible and can also be used as thickening agents. The curing step was performed directly after dip-coating. Following a short-time irradiation of UV light, the membrane solidification immediately and becomes tough. Subsequently the organic part provided the strength of the green membrane was directly removed through an appropriate thermal treatment prior to sintering. The mechanism of UV curing technique in preparation of alumina ceramic membrane is studied and the effects of content of photosensitive resin, firing temperature and drying method on the pore size distribution, water permeation and microstructure of membrane are also discussed. Compared with the traditional preparation process, this method has considerable advantages that eliminating the high shrinkage and cracking risk caused by solvent evaporation while shortening the preparation cycle greatly. This method appeared promising for the preparation of a high-quality and cost-effective ceramic membrane.

## 2. Experimental

### 2.1 Materials

**Fig. 1:**
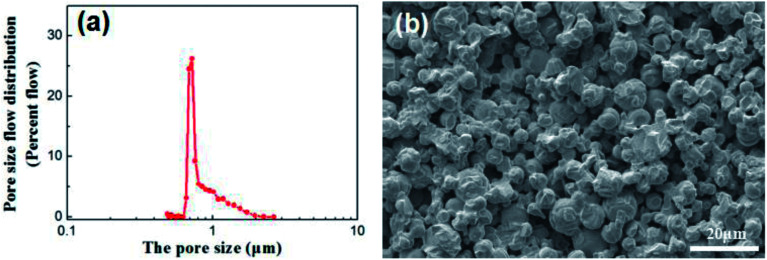
Pore size distribution (a) and cross-section morphology (b) of the disc alumina support.

Disc alumina supports with the sizes of 25 mm in diameter and 1.5 mm in thickness were pre-treated in a 5% hydrochloric aid solution and followed by the thermal treatment at 600 °C for 1 h. The disc supports were stored in a vacuum dryer and purged with nitrogen before use. The characteristics of the disc alumina supports are listed in Table 1, while the corresponding pore size distribution and microstructure are presented in Fig. 1.

**Table 1:** Characteristics of the commercial tubular alumina support

Materials	Linear shrinkage (%)	Open porosity (%)	Average pore size (μm)	Bending strength (MPa)	Water permeation (L m^−2^ h^−1^ bar^−1^)
Alumina	10.45	40.62	0.8	44.9	15 800

Commercially available alpha alumina powder (TM-DAR, *d*_50_ = 0.169 μm, purity 99.99%, Taimei Chemicals Co. Ltd. Nagno-ken, Japan) was used as the main membrane-forming materials. The photosensitive resin (Mitsui Chemicals Group, Japan), an polyurethane acrylate, was dispersed in water of 50 wt% in content and used as the monomers. For the membrane curing with photopolymerisation, the photo photoinitiator Irgacure®1173 (BASF Co. Ltd. Germany) was used. This liquid photoinitiator was made from 2-hydroxy-2-methyl-1-phenyl-1-propanone. Polyvinyl alcohol (PVA) (AH26, Sinopharm Chemical Reagent Co., Ltd, China) was applied as the membrane-forming additives and pore former. Glycerol (C_3_H_8_O_3_, Shanghai Richioint Chemical Reagents Co., Ltd, China) was used as the plasticiser and an anionic surfactant (Hypermer KD-1; Croda, UK) as the dispersant. Deionized water (18.2 MΩ) was used in all preparation processes.

### 2.2 Fabrication of alumina ceramic membrane

In accordance to the formulas in Table 2, the alumina powder, the monomers, the photoinitiator and the dispersant were added to a planetary mill jar and milled for 60 min. Then PVA and glycerol were added to this mixture and stirred for another 30 min. The slurry was treated for 1 min with a homogenous emulsifying machine (IKA T18, Germany) at a speed of 8000 rpm to break the agglomeration of nano-alumina particles. The as-obtained ceramic slurry was degassed at room temperature under the vacuum degree of 3 kPa for beyond 30 min. A uniform coating suspension was obtained.

**Table 2:** The formula of suspension of UV curing

Formula name	Al_2_O_3_ (g)	Monomers (g)	Photoinitiator (mg)	PVA (g)	Glycerol (g)	Dispersant (g)	Deionized water (g)
S5	20.00	1.00	0.01	1.00	0.80	0.20	76.99
S10	20.00	2.00	0.02	1.00	0.80	0.20	75.98
S15	20.00	3.00	0.03	1.00	0.80	0.20	74.97
S20	20.00	4.00	0.04	1.00	0.80	0.20	73.96
S25	20.00	5.00	0.05	1.00	0.80	0.20	72.95
S25′	20.00	5.00	0.00	1.00	0.80	0.20	73.00
S30	20.00	6.00	0.06	1.00	0.80	0.20	71.94

The forming of the green membrane follows: the support, of which one side had been covered with adhesive tape, was dip coated in the slurry for 16 s at a withdrawal speed of 5 mm s^−1^. With a high pressure mercury lamp, the undried membranes were directly exposed to the light source for 30 s (RX 2 kW, 400 mm, Dongguan Ergu Photoelectric Technology Co., Ltd). Subsequently to curing, the sample was placed in an oven for drying at 150 °C. Following drying, the green membranes were moved into an electric furnace and fired according the temperature schedule: from room temperature to 600 °C, the temperature was increased at a rate of 2 °C min^−1^ and soaked at 600 °C for 1 h to remove the organic matters, after that it was raised to the firing temperature at a rate of 5 °C min^−1^ and held for 2 h at firing temperature.

### 2.3 Measurement methods

The hardness of the green membrane was measured with the pencil scratch tester (QHQ-A, China). The weight loss test of the wet membrane is performed by the wet film prepared on the glass substrate, which was peeled from the porous support, to avoid interference caused by solvent evaporation in the support. The thermal analysis of the wet membrane was done by a thermal analyzer (Netzsch STA 449C, Germany). The absorbance spectra were measured with the Lambda 950/UV/Vis/NIR spectrophotometer (Perkin-Elmer, America). The morphology of membrane was observed by a SEM (ZEISS EVO 18, Germany). Fourier Transform Infrared Spectroscopy (FTIR) spectra were recorded using a VERTEX 70 (Bruker, Germany), in Attenuated Total Reflection (ATR) mode. The water permeance of the membrane was measured with the Fully Automated Fluid and Gas Handing Systems OSMO Inspector 2.0 (Poseidon, Convergence, Netherlands). The membrane permeance was determined by collecting the permeation in a mass flowmeter of the OSMO Inspector and timing the collection period. To avoid non-stationary transient effects, the membranes were saturated with deionized water (18.2 MΩ) before the pressure was applied. The effective filtration area of each sample was 2.64 × 10^−4^ m^2^ by measurements and calculations.

The pore size distribution of the disc alumina support and the prepared membrane were characterized with a capillary flow porometer (Porolux 500, IB-FT GmbH, Germany). The membranes were fully wetted with the commercial low surface tension liquid Porefil (surface tension 16 dyn cm^−1^). The measurements consisted of a wet-run program and a dry run program, all the measurements were carried out following the procedure described in literature.^21^ The mean pore radius *r*_min_ (nm) and maximum pore size *r*_max_ (nm) were determined with the computer software.

The rejection performance of the membrane was tested through different sizes of monosize PS microspheres filtering in aqueous dispersion with the concentration of 100 mg L^−1^. To drive the flux through the membrane, a tangential filtration described further was applied to the permeated fluid to maintain a transmembrane pressure of 1 bar and the cross flow velocity at 0.9 m s^−1^. The permeate concentration was determined with the Lambda 950/UV/Vis/NIR spectrophotometer (Perkin-Elmer, America) and compared to the initial concentration, to obtain the rejection rate.

1 *R* (%) = (1 − *C*_p_/*C*_o_) × 100%

where *R* (%) is the rejection rate, *C*_p_ (mg L^−1^) the permeate concentration, and *C*_o_ (mg L^−1^) the initial concentration.

## 3. Results and discussion

### 3.1 Investigation of UV curing process

**Fig. 2:**
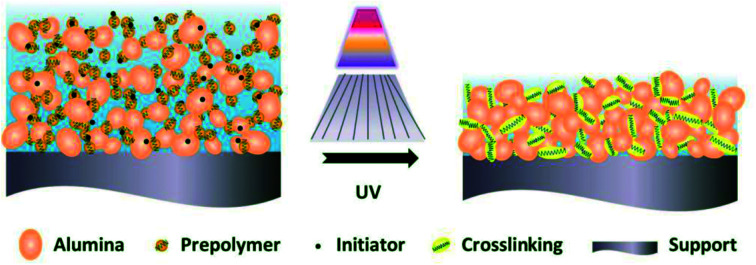
Schematic representation of the crosslinking phenomena of the polymer under UV irradiation.

The polymerization of the green membrane under UV irradiation is illustrated in Fig. 2. The energy produced by the UV light could activate the photoinitiator to generate the free radical and consequently break the C=C bond of the photosensitive resin, for the resin to polymerize rapidly with chain reactions, forming a strong network within a few seconds to a few tens of seconds in a general system.^19^ This polymerization led to a rapid hardening of the green membrane.

**Fig. 3:**
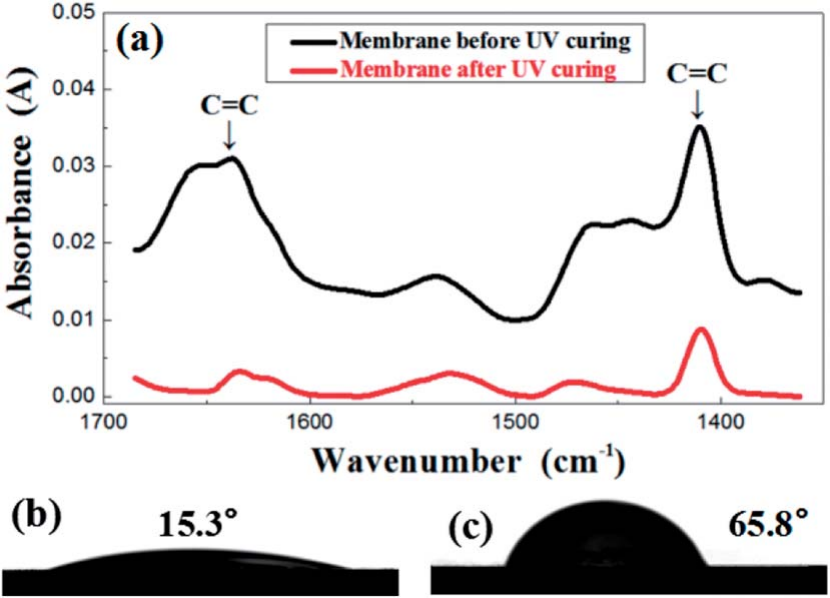
FTIR spectrum (a) before and after UV irradiation of the green membrane using formula S25; contact angle before (b) and after (c) UV irradiation of the green membrane using formula S25.

In order to investigate the change of membrane composition during UV curing, FTIR was performed and the results are presented in Fig. 3a. The evolution of the FTIR spectra was recorded before and after the irradiation under UV light. The carbon–carbon double bond of the resin was characterized by the peaks in the region of 1600–1660 cm^−1^, relevant for the C=C stretching as well as the region of 1400–1430 cm^−1^, representative of the twisting. The absorbance value of those two peaks obviously decreased after UV treatment, which indicates a sharp reduction in the number of double bonds after an exposure. This indirectly reflects that the crosslinking reaction did occur in the membrane. Corresponding to the transformation of structure, the contact angle also demonstrated the great difference before and after UV treatment, shifting from 15.3° to 65.8°, as is shown in the Fig. 3b and c.

Another intuitive property change is hardness of the membrane measured by standard pencil hardness tester. As is shown in Table 3, within 10 s of the initial exposure, the hardness of the membrane using formula S25 did not have a significant change. At this point, the photoinitiator was not able to generate sufficient radicals to break the C=C bond in a relatively short irradiation time, which greatly influences the crosslinking degree of green membrane. Actually, in the 10–20 s, the hardness has a rapid improvement, from 1H directly up to nearly 3H. After 20 s, an increase in hardness gradually slowed down and eventually stayed within 3H. Therefore the curing time was set to 30 s.

**Table 3:** Pencil harness of green membrane as a function of exposure time

Exposure time (s)	10	15	18	20	25	30	40	50	60
Pencil hardness (H)	0	1–2	2	2–3	2–3	3	3	3	3

In order to evaluate the effect of thermal radiation during UV irradiation on the consolidation of wet membrane, the membrane without photoinitiator (formula S25′) was also treated under the same process as the one mentioned above. The results showed that the wet membrane without photoinitiator did not have any strength after UV irradiation of 30 s, which indicated that UV curing played a key role in the hardening of the wet membrane.

**Fig. 4:**
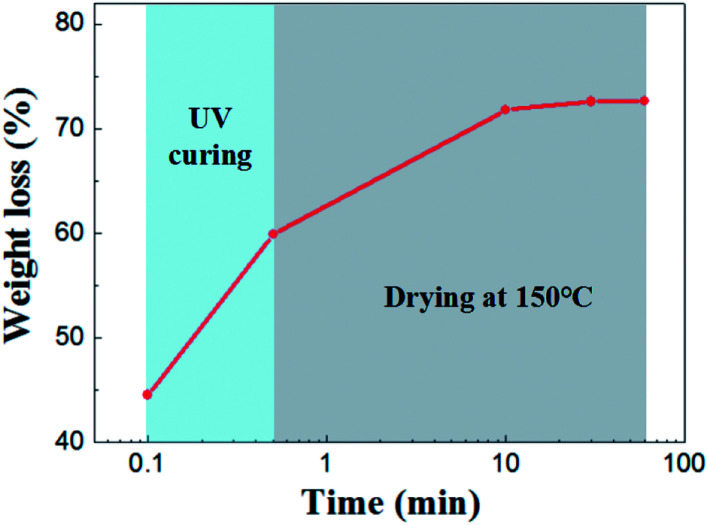
Weight loss as a function of drying time for the wet membrane.

Drying behavior of the UV curing membrane was studied. As for the supported membrane, drying occurs from the top surface and there is no noticeable difference in the drying behavior of wet membrane coated on porous support or nonporous substrate.^22,23^ Therefore it was characterized by taping casting membrane on glass substrate. As is shown in Fig. 4 shows, the initial weight loss of solvent was 44.56%. This part of weight loss is due to the rapid absorption of solvent in the slurry by the pore of support under capillary force. Subsequently, 14.67% of solvent was lost in the process of UV curing. After 30 minutes of drying at 150 °C, almost all the solvent were removed from the membrane.

**Fig. 5:**
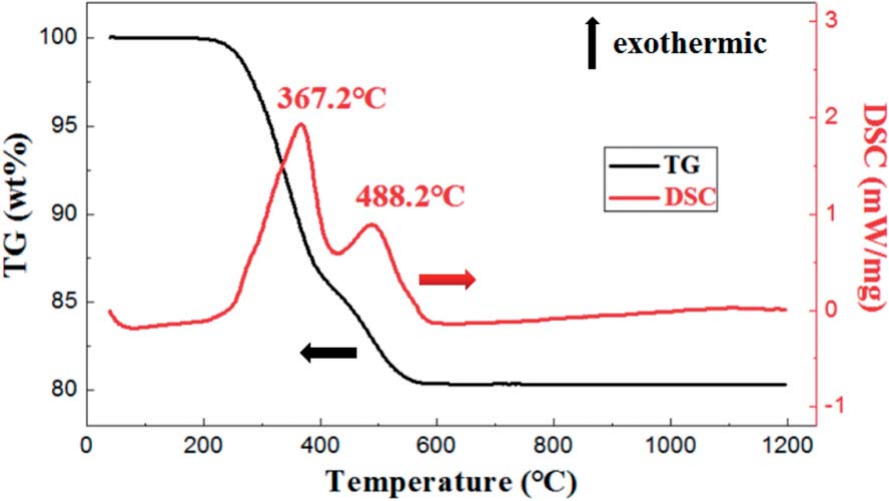
DSC-TG analysis of membrane with weight ratio of prepolymer/Al_2_O_3_ of 25 wt%.

As is shown in Fig. 5, the exothermic loss of about 2.0 wt% in the range of 150–250 °C was attributed to the decomposition of polyvinyl alcohol. The drastic weight loss occurred at temperatures from 300 °C to 500 °C was mainly due to the decomposition of photosensitive resin. Besides, thermal analysis showed that organic matter was almost removed before 650 °C.

### 3.2 Optimization of membrane structure and properties

Based on the method of UV curing, the content of photosensitive resin cured components (hereinafter referred to as prepolymer) and the sintering process were both controlled in order to prepare alumina micro filtration membranes with better performances (Table 4).

**Table 4:** Pencil harness of green membrane as a function of weight ratio of prepolymer/Al_2_O_3_

Weight ratio of prepolymer/Al_2_O_3_ (wt%)	5	10	15	20	25	30
Pencil hardness (H)	0–1	1–2	2	2–3	3	3

**Fig. 6:**
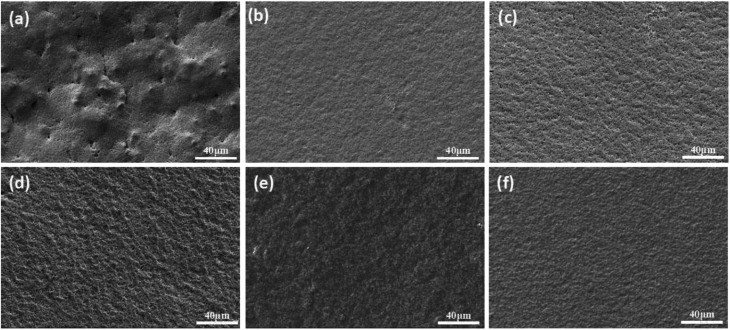
Green membrane cured by UV irradiation with different weight ratio of prepolymer/Al_2_O_3_. (a): 5 wt%; (b): 10 wt%; (c): 15 wt%; (d): 20 wt%; (e): 25 wt%; (f): 30 wt%.

The hardness of the film reflects the integrity of the crosslinking network, and the results show that the content of prepolymer increases as gradually and obviously as the hardness does. However, there is a critical value above which the film hardness no longer depends on the prepolymer content. From the morphology of the cured green membrane characterized by SEM (Fig. 6), cracks in the surface of membranes can be observed when the prepolymer content is 5 wt% (Fig. 6a). While smooth and crack-free membrane is obtained with an increase of prepolymer content, which improves the hardness of the membrane. Hence, the amount of prepolymer has a great influence on UV curing process.

**Fig. 7:**
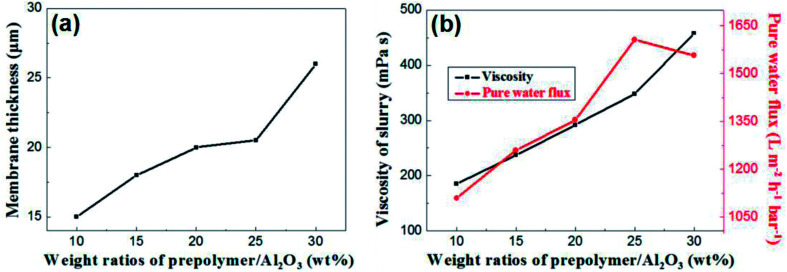
(a): The relation between membrane thickness and weight ratio of prepolymer/Al_2_O_3_; (b): slurry viscosity and pure water flux of membrane as a function of weight ratio of prepolymer/Al_2_O_3_.

The content of prepolymer also affects the viscosity of the membrane-forming suspension and the thickness of the membrane. As shown in Fig. 7a, the membrane thickness increases from 15 μm to 26 μm when the prepolymer/Al_2_O_3_ ratio increases from 5 wt% to 30 wt%.

It is generally known that although the membrane permeation is greatly affected by porosity and thickness, while the viscosity of membrane forming suspension could change both properties in the green membrane. Besides, the higher viscous suspension prevents fine particles from suction into supports, which leads to increase of permeation.^24^ As shown in Fig. 7b, permeance of the membrane increases with weight ratio of prepolymer/Al_2_O_3_ at the range from 5 wt% to 25 wt%, which could be attributed to increment of pore size and porosity. However, there exists a turning point in the curve, presenting that when the ratio was up to 30 wt%, the permeance saw a modest reduce. This is mainly due to a surge in membrane thickness, which surpasses the effect of porosity increment, and the pore size increases slightly.

**Fig. 8:**
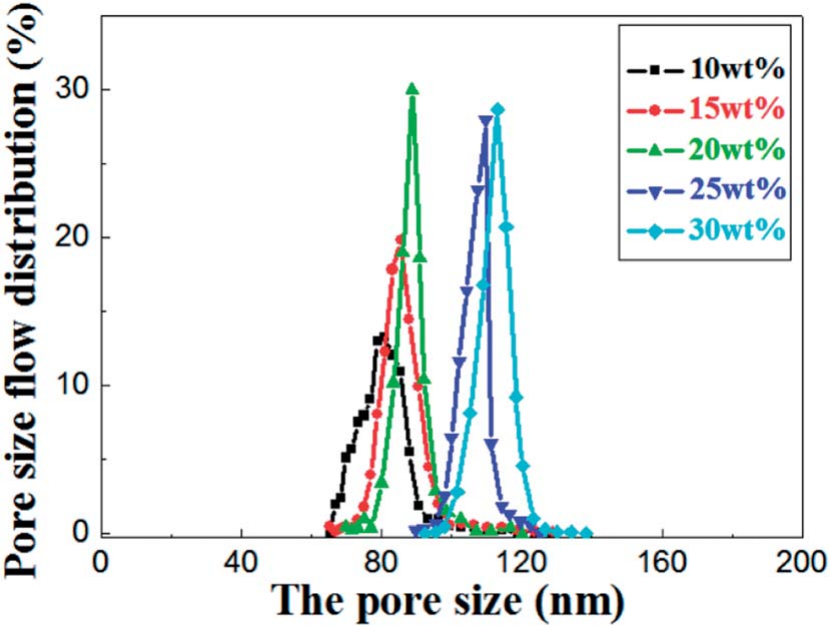
Pore size distribution of the sintered membrane with different weight ratio of prepolymer/Al_2_O_3_ and fired at 1200 °C for 2 h.

As shown in the Fig. 8, the most frequent pore size of the membranes increases with elevation of the relative amount of prepolymer, ranging from 80.9 nm to 113.5 nm and the pore size distribution narrows down gradually. It concludes that UV curable assisted ceramic membranes have a relatively narrow pore size distribution. This is mainly due to the rapid polymerization of prepolymer under UV irradiation. According to Gonzalez,^25^ the adsorption of PVA molecules onto the alumina particles surface reduces their migration in the suspension, which leads to a well dispersion of alumina particles in green membrane before UV cured. The prepolymer in the green membrane is to form a strong network after polymerization, which helps to prevent crack formation from drying shrinkage.

Based on the above analysis, prepolymer content is a key factor in adjusting the suspension viscosity and the thickness of the membrane.

**Fig. 9:**
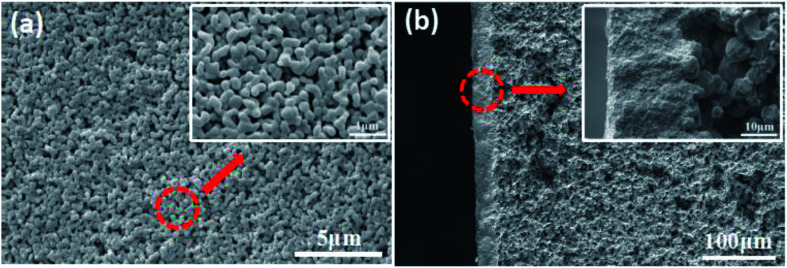
Morphology of the membrane prepared with the prepolymer content of 25 wt% and sintered at 1200 °C. (a): surface; (b): cross section.

The morphology of the sintered membrane characterized by SEM is shown in Fig. 9a, smooth and defect-free membrane with uniformly distributed pores was obtained. The thickness of the membrane is about 20 μm (Fig. 9b). The interface between the separation layer and the support is clearly visible, and the membrane and the support are tightly bonded.

**Fig. 10:**
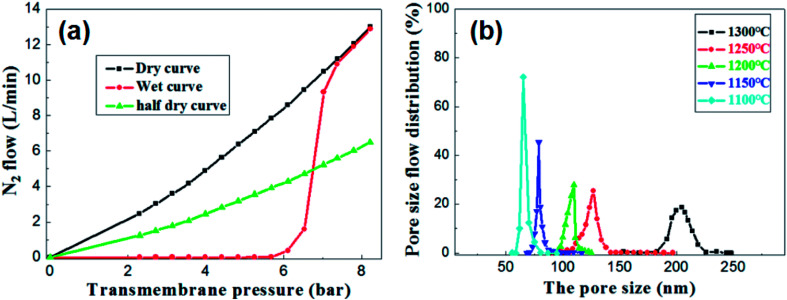
(a) The nitrogen flow rates through the wet and dry membrane with prepolymer/Al_2_O_3_ of 25 wt% and fired at 1100 °C for 2 h; (b) pore size distribution of the membrane with prepolymer/Al_2_O_3_ of 25 wt% and fired at different temperatures.

Under the condition of setting the prepolymer/Al_2_O_3_ ratio to 25 wt%, the influence of firing temperature on the pore size distribution was investigated. The pore size distribution of the membrane fired at 1100 °C for 2 h was measured and calculated through the gas bubble pressure method at 25 °C according to the ASTM F316-03(2011) standard. The relationship between the nitrogen flow and the trans-membrane pressure is shown in Fig. 10a. The gas flow of the wet membrane occurred at the first bubble point of 5.3 bar, which corresponds to the largest membrane pore size of 85.7 nm. As the trans-membrane pressure increased, more pores were opened and the gas flow increased nonlinearly. When the trans-membrane pressure is increased to 7.0 bar, the gas flow increased sharply, indicating that the most frequent pore size of the membrane is about 65.2 nm. After all of the membrane pores are opened, the gas flow increased linearly with the trans-membrane pressure according to the Hagen Poiseuille equation. The calculated pore size distributions of the membranes are shown in Fig. 10b. The largest pore size increases from 85.7 nm to 222.3 nm as the temperature increased from 1100 °C to 1300 °C. Meanwhile the most frequent pore size rises from 65.2 nm to 204.6 nm while the pore size distribution broadens at the same time. The main reason is that the growth of grains leads to the formation of large pores and the elimination of small pores when the membranes are fired at higher temperature.

**Fig. 11:**
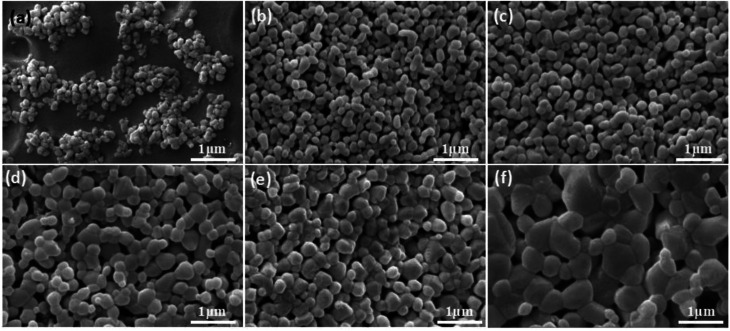
Raw power (a) and membrane fired at different temperatures with prepolymer/Al_2_O_3_ of 25 wt% (b–f). (b): 1100; (c): 1150; (d): 1200; (e): 1250; (f): 1300 °C.

Microstructure of membrane in Fig. 11 demonstrates that there is no obvious change in the grain sizes of the membranes fired below 1150 °C (Fig. 11b and c), compared with the size of raw powder (Fig. 11a). However, as shown in Fig. 11d, the obvious grain growth in the membrane surface fired at 1200 °C has been observed, the size of most grains is located in the range from 300 nm to 500 nm. The change law of membrane pore size is in agreement with the theory of constrained sintering^26,27^ (Fig. 11b–f).

**Fig. 12:**
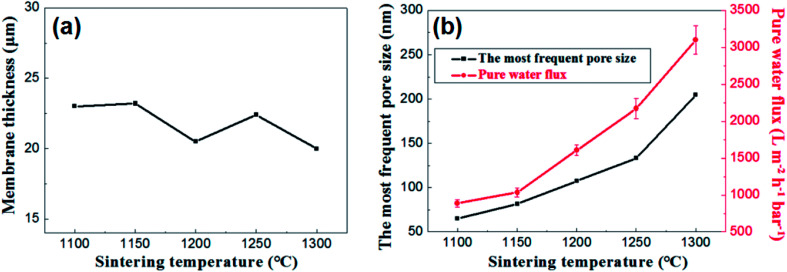
(a): The relation between membrane thickness and sintering temperature; (b): the most frequent pore size and pure water flux of membrane as a function of sintering temperature.

Fig. 12 presents the effects of firing temperature on the membrane thickness, the most frequent pore size and the water permeance of the membrane. As is shown in the Fig. 12a, no great changes occur in the thickness of the membranes at different firing temperature ranging from 20 μm to 23 μm. While the change in flux is still a good illustration of the change in pore size distribution (Fig. 12b).

Based on the above analysis, the firing temperature controlling is an effective method to regulate the pore size distribution and water permeance of the membrane on large-scale. It is suitable to prepare narrow pore size distribution and high flux alumina ceramic membrane at a relative low firing temperature.

### 3.3 Controlling of pore size distribution of membrane

**Fig. 13:**
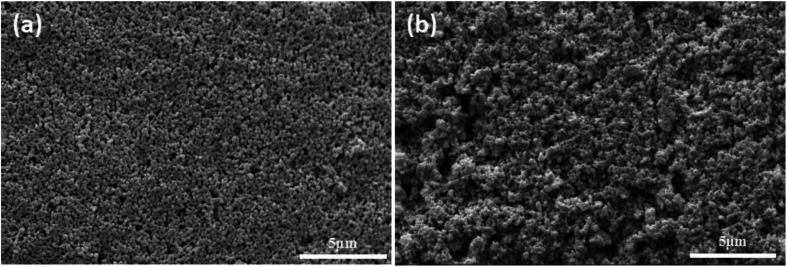
Surface of membrane using different drying method and sintered at 1100 °C for 2 h. (a): UV curing; (b): heat curing.

In order to illustrate the advantage of UV curing process in preparation of alumina ceramic membrane. The green membrane without photoinitiator was used as contrast samples (formula S25′) which had been prepared under the same process of UV curing membrane. The surface morphology of sintered membrane with and without photoinitiator was presented in Fig. 13, the agglomeration of particles clearly occurs in the contrast sample which leads to the appearance of pinholes. On the contrary, smooth and crack free membrane surface with uniformly distributed pores can be observed in the fired UV curing membrane, which indicates that the crosslinked networks play a critical role in resistance to alumina agglomeration and crack formation during the drying process.

**Fig. 14:**
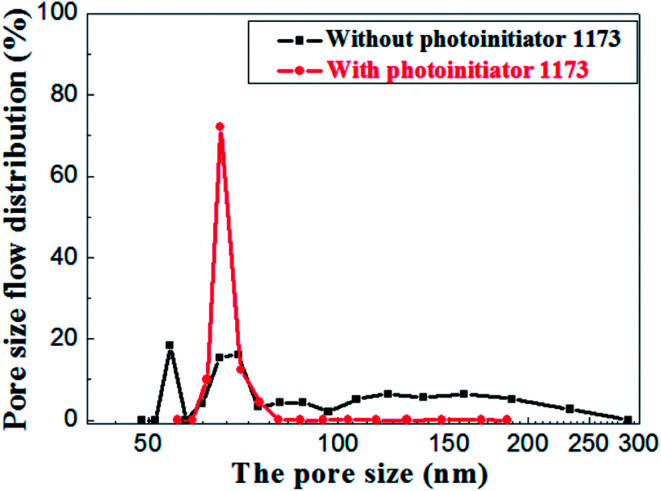
Pore size distribution of membrane with and without photoinitiator.

The pore size distributions of the membranes prepared with and without photoinitiator are shown in Fig. 14, which illustrates the apparent difference between them. The pore size of the membrane prepared without photoinitiator presents a wide distributions with several peaks, but the membrane prepared with UV curing technology presents a sharp pore distribution curve.

Hence, we believe that the macromolecular network formed by rapid crosslinking under UV curing provides toughness to green membrane which effectively resists the tress resulting from dehydration shrinkage. Different from the traditional method, this is a new one that a crosslinking network is formed to prevent the volume effect after drying. Therefore, the drying process of UV curing membrane is much safer and faster than the traditional process.

### 3.4 Characterization of filtration properties

To evaluate the separation performance of the alumina ceramic membrane prepared by UV cured process, the TiO_2_ (80 nm, rutile) slurry with concentration of 100 mg L^−1^ and the membrane with a water permeance of 887 ± 48 L m^−2^ h^−1^ bar^−1^ and the most frequent pore size of 65.2 nm were used. Filtration tests of TiO_2_ slurry were performed under the conditions of temperature at 25 °C, trans-membrane pressure of 1 bar and cross-flow velocity of 0.9 m s^−1^.

**Fig. 15:**
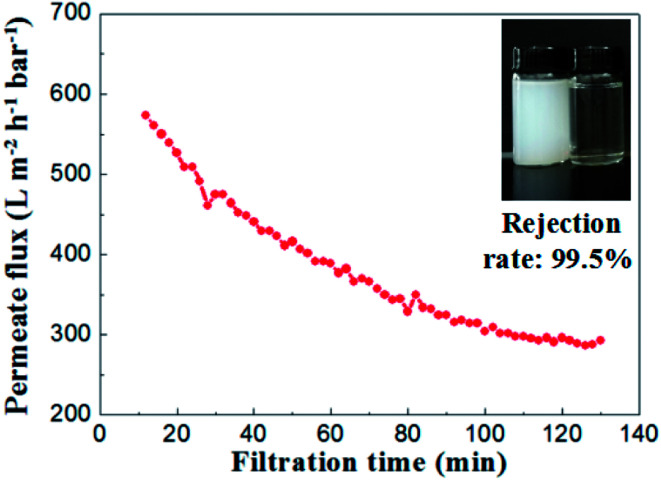
Permeate flux and rejection rate of TiO_2_ emulsion (slurry concentration: 100 mg L^−1^, Δ*P* = 1 bar).

As shown in Fig. 15, the permeate flux of the membrane maintained at a stable value of 300 L m^−2^ h^−1^ bar^−1^ after 100 minutes running. The curve of permeate flux to filtration time might be explained by the formation of a steady cake layer during filtration process which caused the constant resistance permeation of the TiO_2_ slurry.^28^ Meanwhile, the rejection rate remained almost the same (over 99.2%) through the whole filtration experiment. The TiO_2_ contents in the suspension of permeate side were less than 1 mg L^−1^, which indicated well separation performances for the suspension formed by nanoparticles with the size of ∼100 nm.

**Fig. 16:**
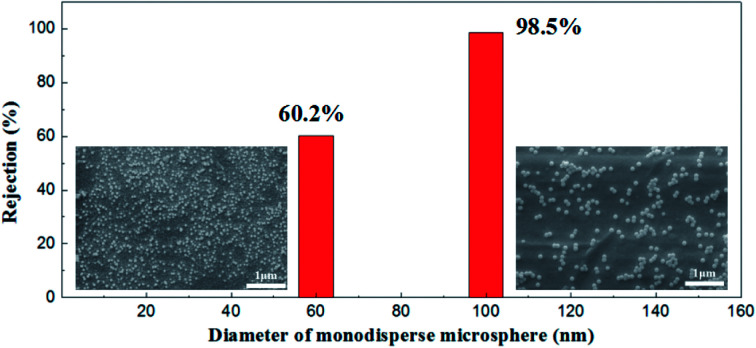
SEM pictures of monosize PS microspheres (diameters of 60 nm and 100 nm) and corresponding rejection performances.

Monodisperse microspheres through dispersion display an extremely narrow particle size distribution, for the microsphere rejection to present a good correspondence with the membrane pore size. For this reason, we can indirectly describe the actual pore size distribution of the membrane by its rejection rate. A 100 mg L^−1^ concentration of microsphere dispersions was prepared with 60 nm and 100 nm in diameters of particles respectively. The rejection performance of the membrane fired at 1100 °C is illustrated in Fig. 16. This membrane could filter out 98.2% of the monosize PS microspheres of 100 nm and 60.1% of the 60 nm monosize PS microspheres, which meant that the membrane had a relative narrow pore size distribution, in accordance with the testing results of the gas bubble pressure method.

To sum up, the UV curing assisted drying method has shown great advantages in the preparation of ceramic membranes. This approach can greatly reduce the drying time and shorten the preparation period while maintaining the superior performance of the ceramic membrane. Its performances are listed with those of other literature as shown in Table 5.

**Table 5:** Comparisons with other literatures

Membrane materials	PWF of membrane (L m^−2^ h^−1^ bar^−1^)	Average pore size (nm)	Membrane thickness (μm)	Drying method of green membrane	Reference
Al_2_O_3_	3101 ± 194	202	22	30 s UV curing and 5 min drying at 150 °C	This work
	1607 ± 75	110	20		
	887 ± 48	69	23		
Al_2_O_3_	2150	200	15	Room temperature for 12 h and at 120 °C for 5 h	2007 (ref. 14)
ZrO_2_	2830	280	10	Room temperature for 12 h and at 120 °C for 5 h	2007 (ref. 15)
TiO_2_	3037	280	40	Room temperature, 70 and 110 °C for 12 h, respectively	2008 (ref. 29)
Al_2_O_3_–TiO_2_	1145	200	20	Drying at room temperature overnight and at 120 °C for 6 h	2009 (ref. 30)
Al_2_O_3_	1000	180	20	Room temperature for 24 h	2011 (ref. 31)
TiO_2_	1150	380	—	60 °C for 24h	2015 (ref. 32)
Attapulgite nanofibers	1540	250	6.7	Dried at room temperature for 24 h, at 70 °C for 12 h, and at 110 °C for 12 h	2015 (ref. 33)
Al_2_O_3_	1400	180	20	Room temperature for 24 h	2016 (ref. 34)
Al_2_O_3_	550	130	15	65 °C for 24h	2017 (ref. 35)
ZrO_2_	2000	200	—	—	Pall. Co. Ltd^36^
	1500	100	—		

## 4. Conclusion

In this work, a rapid preparation of high permeable and reliable alumina membrane has been realized by UV curable technique. The network formed by cross-linking of photocurable resins resulted in rapid solidification of the wet membrane. In this way, the green membrane might obtain its strength at the initial stage of drying and eliminate the drying defects signally. The membrane prepared by this method had a permeance of 887 ± 48 L m^−2^ h^−1^ bar^−1^ when its most frequent pore size was 65.2 nm. The membrane could filter out 98.2% of the monosize PS microspheres of 100 nm and 60.1% of the 60 nm monosize PS microspheres, which meant that a narrow pore size distribution was achieved, corresponding well with the testing results of the gas bubble pressure method. Compared with the uncured method, the UV curing process contributes greatly to avoid particle aggregation and drying defects in drying process. This energy saving and environmental approach could highly reduce the drying time and shorten the preparation period while maintaining the superior performance of the ceramic membrane, which could be universally applied in more industries in the future.

## Conflicts of interest

There are no conflicts to declare.

## Supplementary Material
